# Integrating biomedical HIV prevention in schools: Protocol for a systematic review of interventions for adolescent girls and young women in sub-Saharan Africa

**DOI:** 10.1371/journal.pone.0352973

**Published:** 2026-07-20

**Authors:** Cecilia Afua Lodonu-Senoo, Lily Ogyiri, Kwasi Torpey, Francis Zotor

**Affiliations:** 1 Department of Family and Community Health, Fred N. Binka School of Public Health, University of Health and Allied Sciences, Hohoe, Volta Region, Ghana; 2 Department of Public Health, Robbins College of Health and Human Sciences, Baylor University, Waco, Texas, United States of America; 3 Department of Population, Family and Reproductive Health, School of Public Health, University of Ghana, Accra, Greater Accra Region, Ghana; The University of Iowa College of Public Health, UNITED STATES OF AMERICA

## Abstract

**Introduction:**

Despite global progress in reducing HIV incidence, adolescent girls and young women in sub-Saharan Africa remain disproportionately affected. Biomedical HIV prevention tools such as pre-exposure prophylaxis, condoms and HIV testing are effective but underutilized, especially within facility-based models. Schools represent consistent and scalable contact points for reaching adolescent girls and young women, yet evidence on the feasibility, accessibility and effectiveness of school-based biomedical HIV prevention interventions remains limited.

**Objective:**

This systematic review aims to synthesize evidence on how school-based programs implement, deliver and achieve outcomes in HIV prevention interventions involving pre-exposure prophylaxis, condom and HIV testing for adolescent girls and young women in sub-Saharan Africa.

**Materials and methods:**

This review will follow the Preferred Reporting Items for Systematic Reviews and Meta-Analyses Protocols guidelines. Eligible studies will include randomized and non-randomized trials and qualitative research published between January 2015 and December 2025. Databases to be searched include PubMed, Embase, PsycINFO, CINAHL and the African Index Medicus, together with relevant grey literature sources (WHO, UNAIDS, UNICEF and PEPFAR). Two reviewers will independently screen titles, abstracts, and full texts, extract data, and assess risk of bias using appropriate tools. Quantitative data will be synthesized using meta-analysis where feasible, while qualitative findings will undergo thematic synthesis. Outcomes of interest include the feasibility, accessibility, and effectiveness of school-based HIV prevention interventions.

**Discussion:**

This review will provide a comprehensive synthesis of evidence on biomedical HIV prevention interventions delivered in school settings for adolescent girls and young women in sub-Saharan Africa. Findings will highlight implementation process, barriers, facilitators and equity considerations.

Trial registration number: PROSPERO CRD420251249595

## Introduction

Global progress in human immunodeficiency virus (HIV) prevention has accelerated in recent years, with the year 2024 recording a 40% reduction in incidence compared to 2010. However, an estimated 1.3 million people acquired HIV in 2024, nearly three times the global target of fewer than 370,000 new infections by 2025 [1]. Sub-Saharan Africa accounted for half of all new infections, even as the region experienced the steepest decline globally, a 56% decrease in incidence over the same period [1].

Adolescent girls and young women (AGYW) remain disproportionately affected. HIV incidence among AGYW is more than three times higher than that of their male peers [1]. Although global targets aimed to reduce new infections among AGYW to less than 50,000 by 2025, an estimated 210,000 AGYW acquired HIV in 2024 [1]. In eastern and southern Africa, the estimated number of new HIV infections among AGYW was 140,000, and in western and central Africa it was 31,000 [1]**.** This persistent vulnerability reflects deep-rooted gender inequalities, power imbalances, restrictive social norms, stigma, discrimination, criminalization, violence and socioeconomic inequalities across sub-Saharan Africa, which collectively limit AGYW’s ability to negotiate safer sex, access services, and exercise sexual autonomy [1,2].

Despite the availability of effective prevention tools, uptake is inadequate. Condom use, one of the most cost-effective HIV prevention strategies, remains suboptimal due to stigma, limited access, gendered power dynamics, and scarcity of youth-friendly service environments [3–5]. Likewise, biomedical HIV preventions options such as pre-exposure prophylaxis (PrEP), condom distribution and HIV testing, have expanded the prevention landscape, yet their uptake among AGYW is suboptimal, especially within traditional facility-based models [1].

Schools represent one of the most consistent and scalable contact points for reaching AGYW in sub-Saharan Africa. They offer access to large cohorts of adolescents in a structured setting and can serve as effective entry points for integrated sexual and reproductive health interventions. Evidence demonstrates that educational programmes can enhance HIV-related knowledge, build agency, and promote protective behaviours among adolescents [6,7]. However, curriculum-based HIV education alone has shown limited effectiveness in reducing HIV incidence or maintaining behaviour change without complementary access to prevention services [8,9].

Emerging research suggests that schools may also be promising delivery sites for biomedical HIV prevention interventions such as PrEP, condom distribution, and HIV testing. Studies have reported higher PrEP uptake in school-based or school-linked delivery models compared to community or clinic-based approaches [10]. Qualitative research further indicates that comfort, accessibility, trust, and peer support facilitate PrEP initiation among AGYW in school environments [11]. This suggests that integrating biomedical prevention within school settings may help overcome many of the barriers that limit AGYW’s access to facility-based services.

Existing reviews have focused largely on sexual and reproductive health education, abstinence-only, or broader behavioural interventions, with limited attention to school-based biomedical or service-oriented HIV prevention initiatives and their implementation outcomes [8,12–14].

This systematic review will address this gap by synthesizing quantitative and qualitative evidence to understand what works, what is feasible, and what barriers and facilitators influence the implementation of school-based HIV prevention initiatives, including PrEP, condom, and HIV testing for AGYW in sub-Saharan Africa. The findings will inform programme design, implementation, and scale-up of efforts aimed at reducing HIV incidence among AGYW.

### Objective

To synthesize evidence on how school-based programs are implemented, delivered, and achieve outcomes in HIV prevention interventions (involving PrEP, condom and HIV testing) for AGYW in sub-Saharan Africa.

#### Review questions.

1. What is known about the feasibility of implementing PrEP, condom or HIV testing interventions in school settings in sub-Saharan Africa?
2. What evidence exists on the accessibility of such interventions among AGYW?
3. How effective are these interventions at improving HIV prevention outcomes?

## Materials and methods

This protocol was developed using the Preferred Reporting Items for Systematic reviews and Meta-Analyses Protocols (PRISMA-P) checklist.

### Eligibility criteria

For this review, biomedical HIV prevention interventions refer to school-based interventions that provide, deliver, or facilitate access to PrEP, condoms, or HIV testing services.

Selected studies will be based on the eligibility criteria outlined in Table 1:

**Table 1:** Study eligibility criteria.

Component	Include	Exclude
**Population**	AGYW, aged 10–24 years, enrolled in schools in sub-Saharan Africa	Studies focused exclusively on boys or adult women, Out-of-school youth Studies not disaggregating AGYW data Studies conducted outside sub-Saharan Africa
**Intervention**	School-based HIV prevention programs that include at least one of the following: PrEP education/delivery, condom promotion/distribution, or HIV testing/counselling (in-school or linked to external services)	No intervention, Interventions delivered solely in community or clinic settings without school linkage Interventions limited to general life skills or abstinence-only programs without biomedical or service components.
**Outcomes**	Feasibility, accessibility, effectiveness	Studies reporting only process indicators without implementation or effectiveness outcomes
**Study design**	Randomized-controlled trials, Cluster-randomized trials Quasi-experimental studies, Cohort/longitudinal, implementation studies, mixed-methods studies, qualitative studies	Editorials, commentaries, reviews
**Context**	Formal educational institutions (e.g., public, private, faith-based, vocational schools) in sub-Saharan Africa	Non-school settings (e.g., community centres, Clinics, households)
**Time Frame**	Studies published between January 2015 and December 2025	Studies published before 2015
**Language**	English	Non-English publications

### Information sources

#### Search strategy.

We will search multiple online databases to identify peer-reviewed articles and grey literature. The following databases will be searched:

- PubMed through the PubMed interface using MeSH terms and keywords
- Embase via Elsevier using Emtree Terms and keywords
- PsycINFO via EBSCOhost using APA Thesaurus and keywords
- CINAHL via EBSCOhost using CINAHL Subject Headings and keywords
- African Index Medicus via WHO AIM portal using keywords
- Grey literature: WHO, UNAIDS, UNICEF, PEPFAR

The search will be restricted to studies published between January 2015 and December 2025. The year 2015 was selected because it coincides with major developments in global HIV prevention policy and programming. In 2015, the United Nations adopted the Sustainable Development Goals (SDGs), which included a commitment to end the AIDS epidemic as a public health threat by 2023. That same year, the World Health Organization (WHO) recommended the broader use of oral PrEP for populations with substantial risk of HIV infection [15,16]. Restricting the review to studies published between 2015 and 2025 ensures that the evidence reflects contemporary HIV prevention policies, service delivery models, and implementation approaches. Due to resource constraints and the absence of multilingual screening and data extraction capacity, the review will be limited to studies published in English. Table 2 presents an example of the search strategy to be used in PubMed.

**Table 2:** Search strategy in PubMed.

Search	Search term
1	“Schools” [MeSH] OR school-based [tw] OR school* [tw] OR education* [tw] OR classroom*[tw] OR academic* [tw] OR campus* [tw] OR secondary school [tw] OR high school [tw]
2	“HIV infections/prevention and control” [MeSH] OR “HIV Testing” [MeSH] OR “Condoms” [MeSH] OR “Pre-Exposure Prophylaxis” [MeSH] OR “Sexual Health/education” [MeSH] OR “Student Health Services” [MeSH] OR HIV prevention[tw] OR HIV testing[tw] OR PrEP [tw]
3	“Adolescent” [MeSH] OR “Young Adult” [MeSH] OR “Female” [MeSH] OR “Students” [MeSH] OR adolescent girls[tw] OR young women [tw] OR AGYW [tw] OR teen* [tw] OR youth [tw] OR student* [tw]
4	“sub-Saharan Africa”[tw] OR “Africa South of the Sahara” [MeSH] OR Angola [tw] OR Benin[tw] OR Botswana [tw] OR “Burkina Faso”[tw] OR Burundi [tw] OR Cameroon [tw] OR “Cape Verde” [tw] OR Cabo Verde[tw] OR “Central African Republic” [tw] OR Chad [tw] OR Comoros [tw] OR Congo [tw] OR “Democratic Republic of the Congo” [tw] OR Djibouti [tw] OR “Equatorial Guinea” [tw] OR Eritrea [tw] OR Ethiopia [tw] OR Gabon [tw] OR Gambia [tw] OR Ghana [tw] OR Guinea [tw] OR “Guinea Bissau” [tw] OR “Ivory Coast” [tw] OR “Cote d’Ivoire” [tw] OR Kenya [tw] OR Lesotho [tw] OR Liberia [tw] OR Madagascar [tw] OR Malawi [tw] OR Mali [tw] OR Mauritania [tw] OR Mauritius [tw] OR Mozambique [tw] OR Namibia [tw] OR Niger [tw] OR Nigeria [tw] OR Rwanda [tw] OR “Sao Tome” [tw] OR Senegal [tw] OR Seychelles [tw] OR “Sierra Leone” [tw] OR Somalia [tw] OR “South Africa” [tw] OR “South Sudan” [tw] OR Sudan [tw] OR Swaziland [tw] OR Eswatini[tw] OR Tanzania [tw] OR Togo [tw] OR Uganda [tw] OR “Western Sahara” [tw] OR Zaire [tw] OR Zambia [tw] OR Zimbabwe [tw]
5	#1 AND #2 AND #3 AND #4
6	#5 Limits: 01/01/2015 to 31/12/2025, studies in English

### Study records

#### Data management.

All records identified through database searches will be imported into Zotero for reference management. Full-text articles will be downloaded and stored locally, organized by first author surname and year of publication. Extracted data, reviewer decisions, and synthesis outputs will be archived across platforms to ensure transparency and reproducibility.

#### Study selection and data extraction.

Title/abstract screening and full-text review will be conducted using Rayyan, a web-based tool designed to facilitate blinded and collaborative screening. Two reviewers will independently screen each record against the predefined eligibility criteria. Disagreements will be resolved through discussion or adjudication by a third reviewer. Full-text screening will follow the same dual-review process within Rayyan, with exclusion reasons documented systematically.

Studies meeting the eligibility criteria will undergo data extraction, which will be conducted independently by two reviewers using a structured template aligned with the review’s objectives. Conflicts will be discussed and resolved between the two reviewers, and where resolution cannot be reached, a third reviewer will be involved to decide. RevMan will be used to facilitate data extraction, risk of bias assessment and data synthesis.

The reference lists of all included studies will be screened to identify additional eligible studies not captured through the database search.

**Fig 1:**
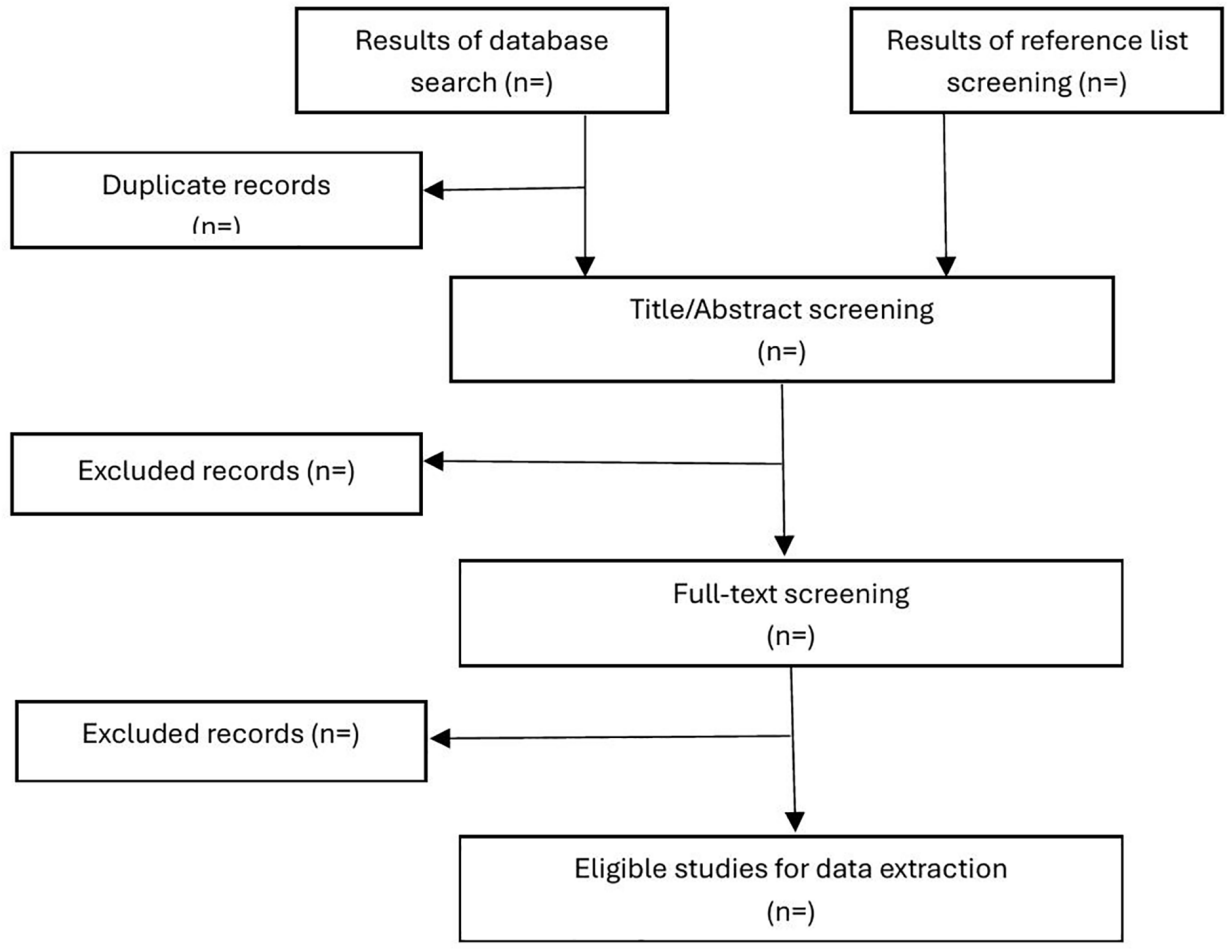
PRISMA flow diagram.

When relevant data are missing or unclear, the corresponding author of the article will be contacted to request additional information. The study selection process will be reported using the PRISMA flow diagram (Fig 1).

#### Data items.

Implementation outcomes will be assessed according to the implementation science frameworks by Proctor and colleagues [17] and adapted to the school-based HIV prevention context. For this review:

1. Feasibility refers to the extent to which the intervention can be delivered within school settings as intended, including logistics, staffing, training, fidelity, and integration into school routines.
2. Accessibility refers to the degree to which AGYW can access and use the intervention, including acceptability, uptake, privacy, confidentiality, perceived appropriateness, and equity of access.
3. Effectiveness (implementation-linked) refers to biomedical or behavioural outcomes directly related to intervention delivery, such as HIV testing uptake, PrEP initiation or continuation, and condom uptake or use. These outcomes reflect real-world effects of implementation rather than clinical efficacy.
4. Secondary implementation outcomes include cost and cost-effectiveness, sustainability or maintenance, engagement of school personnel or parents, and contextual barriers and facilitators such as gender norms, stigma, or school policies.

The list of variables to be extracted and their definitions are presented in Table 3.

**Table 3:** List and definitions of all variables for which data will be sought.

Variable	Definition
***Characteristics of the study***	
Authors	First author and co-authors
Year	Year of publication
Country/region	Country or region within sub-Saharan Africa
Design	Study design
Setting	Type of school or educational institution
Sample size	Number of participants or clusters
Duration	Length of follow-up or intervention period
Funding source	Private sector or Government
***Population and Implementation Variables***	
Population	Adolescent and young women in sub-Saharan Africa
Age range	Specific age range of participants included in the study
Intervention	Type of intervention: condom-related, PrEP-related, HIV testing, or linkage to external services
Comparator	Standard curriculum, community-only interventions, no intervention, other school-based strategies
Delivery personnel	Who implemented the intervention (teachers, health workers, etc)
Mode of delivery	Format of intervention delivery
Engagement	Level of involvement of school staff, parents, community leaders or AGYW themselves
Barriers and facilitators	Contextual factors influencing implementation (e.g., gender norms, power dynamics, structural factors)
***Main Outcome variables (high priority)***	
Feasibility	Implementation, logistics, training fidelity to protocol
Accessibility	Acceptability, uptake, equity, privacy
Effectiveness	Biomedical/behavioural outcomes like HIV testing uptake, PrEP initiation, condom use
***Additional outcomes (moderate priority)***	
Secondary implementation outcomes	Cost and cost-effectiveness, sustainability/maintenance
Knowledge and awareness	Changes in HIV -related knowledge or awareness, risk perception
Behavioural intentions	Intentions for condom use, PrEP, HIV testing
Adverse events or harm	Reported negative outcomes

### Risk of bias assessment

Quality appraisal for randomized trials will be conducted using the Cochrane Collaboration Risk of Bias (ROB 2) tool, including the extension for cluster-randomized trials. For quasi-experimental trials and cohort studies, the Risk Of Bias In Non-randomized Studies of Interventions (ROBINS-I) tool will be used. The CASP qualitative checklist will be used to appraise qualitative studies, and the Mixed Methods Appraisal Tool will be used for mixed-methods studies. Two reviewers will independently assess risk of bias. Disagreements will be resolved through discussion, and where consensus cannot be reached, a third reviewer will be consulted.

### Data synthesis

A descriptive synthesis of all included studies, summarizing intervention types, outcomes and other study characteristics will be presented using structured tables and narrative summaries. Quantitative and qualitative data will be analysed separately before integration.

Quantitative findings will be synthesized using meta-analysis where appropriate, or narratively when heterogeneity precludes statistical pooling. Meta-analysis will be conducted for feasibility, accessibility, and effectiveness outcomes when at least two studies report comparable measures using sufficiently similar definitions, and are sufficiently similar in terms of intervention characteristics, participant population, outcome definitions, and study design. Feasibility outcomes may include implementation fidelity, recruitment, retention, and intervention delivery indicators. Accessibility outcomes may include acceptability, uptake, reach, privacy, and equity-related indicators. Effectiveness outcomes may include HIV testing uptake, PrEP initiation or adherence and condom use. Where sufficient data are available, pooled analyses may be conducted for outcomes such as HIV testing uptake, PrEP initiation, condom use, intervention acceptability, intervention uptake and retention in prevention programs. Binary outcomes will be summarized using risk ratios, odds ratios, or risk differences; continuous outcomes using mean differences or standardized mean differences, and time-to-event outcomes using hazard ratios. A random-effects model will be used as a default analytic approach, with heterogeneity assessed using the I^2^ statistic and Chi-square test. Where at least 10 studies contribute data, subgroup analyses will be conducted by intervention type, delivery setting and predefined age groups. Subgroup analyses will be conducted for predefined age groups (10–14, 15–19 and 20–24 years) when sufficient studies report age-disaggregated data, to examine whether implementation outcomes differ across developmental stages. Meta-regression may be used to explore potential sources of heterogeneity. Sensitivity analyses will examine the influence of excluding studies at high risk of bias or those with missing data. If meta-regression is not feasible due to variability in study design or outcome measurement, findings will be presented narratively. Narrative synthesis will involve grouping studies by intervention type and outcome domain and summarizing results thematically.

Qualitative data will be synthesized thematically using inductive and deductive coding. Themes will be organized under feasibility, accessibility, and effectiveness to align with the review’s core domains. Integration will occur at the interpretive level, with narrative juxtaposition of quantitative and qualitative findings to identify convergence, divergence, and contextual insights relevant to implementation.

Publication bias will be assessed using funnel plots and statistical tests for small-study effects (e.g., Egger’s or Begg’s test), where applicable. Quantitative synthesis will be carried out in RevMan, and qualitative synthesis in Excel.

### Confidence in cumulative evidence

Two independent reviewers will use the Grading of Recommendations Assessment, Development and Evaluation (GRADE) framework to determine the certainty of evidence. Conflicts will be resolved through discussion, and a third reviewer will be involved where agreement cannot be reached.

### Ethical considerations

This is a systematic review of published and publicly available literature. Hence, it will not involve primary data collection with human participants and does not require formal ethical approval. All data will be extracted from existing studies, and no individual participant information will be identifiable. This review will be conducted in accordance with PRISMA-P guidelines to ensure transparency, reproducibility, and integrity throughout the process.

### Status and timeline of the study

Table 4 summarizes the planned timeline for screening, data extraction, analysis and expected completion of the study.

**Table 4:** Study timeline.

Review stage	Status at submission	Planned timeline
Protocol development	Completed	Completed prior to submission
Database search	Not yet started	June 2026
Title/abstract screening	Not yet started	June – July 2026
Full-text screening	Not yet started	August 2026
Data extraction	Not yet started	September – October 2026
Risk of bias assessment	Not yet started	September – October 2026
Data analysis and synthesis	Not yet started	November – December 2026
Report writing and dissemination	Not yet started	December 2026 – February 2027

## Discussion

This systematic review will synthesize evidence on school-based HIV prevention interventions targeting AGYW in sub-Saharan Africa. Implementation of these interventions is often shaped by contextual factors, including stigma, policy constraints, and resource availability.

By focusing on studies conducted within educational institutions, this review aims to clarify what works in integrating biomedical HIV prevention into school settings and to clarify implementation bottlenecks and equity gaps. The inclusion of both quantitative and qualitative components will allow for a nuanced understanding of not only outcomes but also implementation processes, barriers and facilitators.

Potential limitations include variability in study design, outcome definitions, and measurement tools, which may limit the feasibility of meta-analysis. Restricting the review to English-language publications may introduce language bias and may result in the exclusion of relevant studies from francophone and lusophone countries in sub-Saharan Africa. This limitation reflects resource constraints, and the findings should be interpreted in light of this.

Ultimately, this review will contribute to the evidence base for equity-driven HIV prevention programming in schools, helping to inform future research, policy, and practice at reducing HIV vulnerability among AGYW in sub-Saharan Africa.

## Supporting information

S1 Table PRISMA-P checklist. (DOCX)
