# Supplementary material for: Integrating biomedical HIV prevention in schools: Protocol for a systematic review of interventions for adolescent girls and young women in sub-Saharan Africa
Source: PLoS One. 2026 Jul 20;21(7):e0352973. doi: 10.1371/journal.pone.0352973 (PMC13384304; doi:10.1371/journal.pone.0352973)
Supplement: Attachment — Submitted filename: Response to Reviewers.docx [file pone.0352973.s004.docx]

**Response to Reviewers**

We sincerely thank the Reviewers for their thoughtful and constructive comments. We appreciate the positive assessment of the relevance and potential contribution of this protocol. We have carefully revised the manuscript to address all comments and believe that these revisions have strengthened the clarity, transparency, and reproducibility of the proposed review. Below, we provide a detailed point-by-point response.

**REVIEWER #1**

**Comment:**
The manuscript presents a generally relevant and timely rationale for examining the integration of biomedical HIV prevention interventions within school settings for adolescent girls and young women, particularly given the ongoing burden of HIV in this population and the importance of school platforms for prevention delivery. However, the justification for the proposed research questions and eligibility criteria would benefit from further refinement. In particular, the inclusion of a broad age range (10–24 years) is not fully aligned with a school-based intervention focus and introduces substantial heterogeneity across distinct developmental, educational, and service-access stages. Early adolescents (10–14 years), mid-to-late adolescents (15–19 years), and young adults (20–24 years) differ meaningfully in terms of school exposure, sexual and reproductive health needs, and relevance to biomedical HIV prevention interventions. As currently framed, the criteria risk conflating populations for whom school-based biomedical integration may be variably applicable or minimally relevant, particularly at the younger end of the spectrum. The authors should either provide a clearer justification for maintaining this broad age range, including a plan for stratified analysis by age/education stage, or consider narrowing the population to better reflect school-attending secondary school-aged adolescents, where the intervention context is most coherent.

**Response:**
We thank the reviewer for this thoughtful observation. We have retained the 10–24-year age range because it aligns with global definitions of adolescents and young people used by WHO and UNAIDS, and because school-based and school-linked HIV prevention interventions in sub-Saharan Africa frequently reach individuals across this span, including early adolescents in upper primary school, secondary school students, and young adults enrolled in post-secondary or vocational institutions.

However, we agree that developmental and educational stages differ meaningfully within this range. To address concerns regarding heterogeneity, we have revised the Data Synthesis section to specify that subgroup analyses will be conducted by age category (10–14 years, 15–19 years, and 20–24 years) where sufficient studies are available. This approach preserves conceptual coherence while allowing us to capture the full range of school-attending populations targeted by HIV prevention programs in the region.

**REVIEWER #2**

**Introduction/Methods**

**Comment 1:**
Some terms such as ‘biomedical HIV prevention’ and ‘implementation outcomes’ could be defined more clearly to improve clarity and help readers better understand the scope of the review.

**Response:**
We appreciate this helpful suggestion. We have clarified these terms within the Methods section. Specifically, we added an operational definition stating that, for the purposes of this review, biomedical HIV prevention interventions refer to school-based interventions that provide, deliver or facilitate access to PrEP, condoms or HIV testing services. We also expanded the description of implementation-related outcomes, including feasibility, accessibility and effectiveness domains to improve clarity regarding the scope of the review.

**Methods**

**Comment 1**:
The outcomes section could benefit from further clarification. While biological and behavioural outcomes appear to be excluded in the eligibility criteria, they are later mentioned under effectiveness outcomes. Clarifying how effectiveness will be assessed would improve consistency.

**Response:**
Thank you for this helpful observation. We agree that the presentation of outcomes in the eligibility table may have created ambiguity. To improve clarity and ensure consistency across sections, we have revised the eligibility criteria by removing the reference to biological and behavioural outcomes from the inclusion table. These outcomes are now described exclusively under the Data Items section, where we provide operational definitions of feasibility, accessibility, and effectiveness. We also added a clarifying statement to specify that biomedical and behavioural measures (e.g., HIV testing uptake, PrEP initiation, condom use) will be assessed as effectiveness outcomes, reflecting real‑world effects of intervention delivery.

These revisions ensure that the eligibility criteria focus solely on the types of outcomes required for inclusion, while the Data Items and Outcomes sections clearly describe how effectiveness will be assessed.

**Comment 2:**
It would be useful to briefly explain the rationale for limiting the review to studies published between 2015 and 2025, particularly whether this relates to important policy developments such as WHO PrEP guideline updates or the SDG era

**Response:**
We have added a justification for the selected timeframe within the Search Strategy section. Specifically, we note that 2015 coincided with the adoption of the Sustainable Development Goals and WHO recommendations supporting broader use of oral PrEP among populations at substantial risk of HIV infection.

**Comment 3:**
The search strategy could be strengthened. Using “Africa” alone may retrieve studies outside sub-Saharan Africa.

**Response:**
We agree and have revised the search strategy accordingly. The standalone term “Africa” has been removed from the search strategy.

**Comment 4:**
The risk of bias section could be strengthened by specifying which appraisal tools will be used for each study design and how disagreements between reviewers will be resolved.

**Response:**
We appreciate this helpful suggestion. The Risk of Bias Assessment section has been expanded to specify the appraisal tool that will be used for each study design. We also clarify that risk-of-bias assessments will be conducted independently by two reviewers, with disagreements resolved through discussion and consultation with a third reviewer when necessary.

**Comment 5:**
The data synthesis section would benefit from a little more detail regarding which outcomes will be pooled, the effect measures to be used, how heterogeneity will be assessed, and under what conditions meta-analysis will be conducted

**Response:**
We appreciate this helpful suggestion and have revised the manuscript accordingly. We have expanded the Data Synthesis section. The revised text now specifies the types of outcomes that may be pooled, including feasibility, accessibility, and effectiveness outcomes; the effect measures that will be used for binary, continuous, and time-to-event outcomes; the methods for assessing heterogeneity using the I² statistic and Chi-square test; and the conditions under which meta-analysis, subgroup analyses, sensitivity analyses, and narrative synthesis will be conducted.

**Comment 6:**
Restricting the review to English-language publications may exclude relevant studies from francophone and lusophone countries within sub-Saharan Africa. The authors may wish to consider including studies published in other languages where feasible, using available translation tools or multilingual support to minimize language bias and improve the comprehensiveness of the review.

**Response:**
We thank the reviewer for this important observation. We acknowledge that restricting the review to English-language publications may result in exclusion of relevant evidence from francophone and lusophone countries in sub-Saharan Africa and may introduce language bias. However, resource constraints and the absence of multilingual screening and data extraction capacity preclude the reliable assessment of non-English studies. We revised the Discussion section to explicitly acknowledge the potential for language bias and its implications for the comprehensiveness of the review.

We thank the Reviewers for their valuable comments. We believe the revisions have substantially strengthened the protocol and satisfactorily addressed the comments raised by the Reviewers.
