# Supplementary material for: Integrating biomedical HIV prevention in schools: Protocol for a systematic review of interventions for adolescent girls and young women in sub-Saharan Africa
Source: PLoS One. 2026 Jul 20;21(7):e0352973. doi: 10.1371/journal.pone.0352973 (PMC13384304; doi:10.1371/journal.pone.0352973)
Supplement: Attachment — Submitted filename: PONE-D-26-18433_reviewed.docx [file pone.0352973.s002.docx]

PLOS One

Integrating biomedical HIV prevention in schools: Protocol for a systematic review of interventions for Adolescent Girls and Young Women in Sub-Saharan Africa

--Manuscript Draft--

| Manuscript Number: | PONE-D-26-18433 |
| --- | --- |
| Article Type: | Registered Report Protocol |
| Full Title: | Integrating biomedical HIV prevention in schools: Protocol for a systematic review of interventions for Adolescent Girls and Young Women in Sub-Saharan Africa |
| Short Title: | Biomedical HIV prevention in schools for Adolescent Girls and Young Women in SubSaharan Africa. |
| Corresponding Author: | Cecilia Afua Lodonu-Senoo, Mphil  University of Health and Allied Sciences Ho, Volta Region, Ghana, GHANA |
| Keywords: | Adolescent girls and young women; HIV prevention; Pre-Exposure Prophylaxis; School-based interventions; Condom use; HIV testing; sub-Saharan Africa |
| Abstract: | Introduction: Despite global progress in reducing HIV incidence, adolescent girls and young women in sub-Saharan Africa remain disproportionately affected. Biomedical HIV prevention tools such as pre-exposure prophylaxis, condoms and HIV testing are effective but underutilized, especially within facility-based models. Schools represent consistent and scalable contact points for reaching adolescent girls and young women, yet evidence on the feasibility, accessibility and effectiveness of school-based biomedical HIV prevention interventions remains limited.  Objective: This systematic review aims to synthesize evidence on how school-based programs implement, deliver and achieve outcomes in HIV prevention interventions involving pre-exposure prophylaxis, condom and HIV testing for adolescent girls and young women in sub-Saharan Africa.  Materials and Methods: This review will follow the Preferred Reporting Items for Systematic Reviews and Meta-Analyses Protocols guidelines. Eligible studies will include randomized and non-randomized trials and qualitative research published between January 2015 and December 2025. Databases to be searched include PubMed, Embase, PsycINFO, CINAHL, African Index Medicus, and ProQuest. Two reviewers will independently screen titles, abstracts, and full texts, extract data, and assess risk of bias using appropriate tools. Quantitative data will be synthesized using meta-analysis where feasible, while qualitative findings will undergo thematic synthesis. Outcomes of interest include the feasibility, accessibility, and effectiveness of school-based HIV prevention interventions.  Discussion: This review will provide a comprehensive synthesis of evidence on biomedical HIV prevention interventions delivered in school settings for adolescent girls and young women in sub-Saharan Africa. Findings will highlight implementation process, barriers, facilitators and equity considerations. |
| Order of Authors: | Cecilia Afua Lodonu-Senoo, Mphil |
|  | Lily Ogyri, PhD,MPH |
|  | Kwasi Torpey, MBChB, FWACP, FGCP, PhD, MPH |
|  | Francis Zotor, PhD, MSc, PGDip |
| Additional Information: |  |
| Question | Response |
| Financial Disclosure  Enter a financial disclosure statement that describes the sources of funding for the work included in this submission. Review the [submission guidelines](http://journals.plos.org/plosone/s/submission-guidelines#loc-financial-disclosure-statement) for detailed | The author(s) received no specific funding for this work. |

| requirements. View published research articles from [PLOS ONE](https://journals.plos.org/plosone/) for specific examples.  This statement is required for submission and will appear in the published article if the submission is accepted. Please make sure it is accurate. |  |
| --- | --- |
| Funded studies Enter a statement with the following details: - Initials of the authors who received each award - Grant numbers awarded to each author - The full name of each funder - URL of each funder website - Did the sponsors or funders play any role in the study design, data collection and analysis, decision to publish, or preparation of the manuscript? |  |
| Did you receive funding for this work? |  |
| Competing Interests  Use the instructions below to enter a competing interest statement for this submission. On behalf of all authors, disclose any [competing interests](http://journals.plos.org/plosone/s/competing-interests) that could be perceived to bias this work—acknowledging all financial support and any other relevant financial or nonfinancial competing interests.  This statement is required for submission and will appear in the published article if the submission is accepted. Please make sure it is accurate and that any funding sources listed in your Funding Information later in the submission form are also declared in your Financial Disclosure statement.  View published research articles from [PLOS ONE](http://journals.plos.org/plosone/) for specific examples. | The authors have no competing interest. |

| NO authors have competing interests Enter: The authors have declared that no competing interests exist. Authors with competing interests Enter competing interest details beginning with this statement: I have read the journal's policy and the authors of this manuscript have the following competing interests: [insert competing interests here] |  |
| --- | --- |
| * typeset |  |
| Ethics Statement  Enter an ethics statement for this submission. This statement is required if the study involved:   - Human participants - Human specimens or tissue - Vertebrate animals or cephalopods - Vertebrate embryos or tissues - Field research   Write "N/A" if the submission does not require an ethics statement.  General guidance is provided below. Consult the [submission guidelines](http://journals.plos.org/plosone/s/submission-guidelines#loc-guidelines-for-specific-study-types) for detailed instructions. Make sure that all information entered here is included in the Methods section of the manuscript. | N/A |

Format for specific study types

Human Subject Research (involving human

participants and/or tissue)

Give the name of the institutional review

board or ethics committee that approved the

study

•

Include the approval number and/or a

statement indicating approval of this

research

•

Indicate the form of consent obtained

(

written/oral) or the reason that consent was

not obtained (e.g. the data were analyzed

anonymously)

•

Animal Research (involving vertebrate

animals, embryos or tissues)

Provide the name of the Institutional Animal

Care and Use Committee (IACUC) or other

relevant ethics board that reviewed the

study protocol, and indicate whether they

approved this research or granted a formal

waiver of ethical approval

•

Include an approval number if one was

obtained

•

If the study involved

non-human primates

,

add

additional details

about animal welfare

and steps taken to ameliorate suffering

•

If anesthesia, euthanasia, or any kind of

animal sacrifice is part of the study, include

briefly which substances and/or methods

were applied

•

Field Research

Include the following details if this study

involves the collection of plant, animal, or

other materials from a natural setting:

Field permit number

•

Name of the institution or relevant body that

granted permission

•

<

strong>Data Availability</strong><br

>

<

br>Before publication, Authors are

required to make fully available and

without restriction all data underlying their

findings. Sharing the data underlying your

findings increases the visibility, credibility,

and impact of your work, while supporting

transparency and future scientific

discovery. Please see our <a

href="https://journals.plos.org/plosone/s/d

ata-availability" target="_blank">PLOS

Data Policy page</a> for detailed

information on this policy.</p>

<

br>A <strong>Data Availability

No datasets were generated. All relevant data will be made available upon study

completion.

| Statement</strong>, detailing where the data can be accessed, is required at first submission. Insert your Data Availability  Statement in the box below.</p>  <br>Please see the <a  href="https://journals.plos.org/plosone/s/s ubmission-guidelines#loc-data-reporting" target="_blank">data reporting</a> of our submission guidelines for instructions on what you need to include in your Data Availability Statement. Examples of acceptable Data Availability Statements are included here for reference:</p>  <ol>  <li>The minimal data set [and accompanying code, where generated] is available at [repository name] via [DOI hyperlink]</li>  <li>No data was generated by this study. The following existing data sources were used: [dataset name] from [dataset location] available via [web address or  DOI hyperlink].</li>  <li>For Study Protocols: No datasets were generated or analysed during the current study. All relevant data from this study will be made available upon study completion.</li>  </ol>  <br><i>PLOS allows rare exemptions to address legal and ethical concerns. If you have legal or ethical restrictions, please use the box below to detail these in full sentences for the Journal team to consider.</i></p>  <br>This statement will be typeset if the manuscript is accepted for publication.</p> |  |
| --- | --- |

Protocol	[Click here to](https://www2.cloud.editorialmanager.com/pone/download.aspx?id=44514276&guid=09fe6af3-3bed-461d-91af-166011391d06&scheme=1)

[access/download;Manuscript;CLS_Protocol_PLOS_v2.docx](https://www2.cloud.editorialmanager.com/pone/download.aspx?id=44514276&guid=09fe6af3-3bed-461d-91af-166011391d06&scheme=1)

1 **Integrating biomedical HIV prevention in schools: Protocol for a systematic**
2 **review of interventions for Adolescent Girls and Young Women in Sub-**

# 3 Saharan Africa

4

5 Cecilia Afua Lodonu-Senoo^1*^, ceciliasenoo@gmail.com
6 Lily Ogyiri^2^, lilyogyiri@gmail.com
7 Kwasi Torpey^3^, ktorpey@hotmail.com
8 Francis Zotor^1α^, fbzotor@uhas.edu.gh

9

10

11	^1^ Fred N. Binka School of Public Health, University of Health and Allied Sciences, Hohoe, Volta 12	region, Ghana

13 ^2^Robbins College of Health and Human Sciences, Baylor University, Waco, Texas, USA
14 ^3^School of Public Health, University of Ghana, Accra, Greater Accra region, Ghana

15

16 * Corresponding author
17 α Guarantor

18

19

## Funding

This systematic review is funded by Hope for Future Generations. The funder has no role in the design and conduct of the study.

**Competing interests** None to declare.

## Data availability

Upon completion of the review, the extracted dataset and analysis files will be made available in supplementary files or upon request to the corresponding author.

# Abstract

**Introduction:** Despite global progress in reducing HIV incidence, adolescent girls and young women in sub-Saharan Africa remain disproportionately affected. Biomedical HIV prevention tools such as pre-exposure prophylaxis, condoms and HIV testing are effective but underutilized, especially within facility-based models. Schools represent consistent and scalable contact points for reaching adolescent girls and young women, yet evidence on the feasibility, accessibility and effectiveness of school-based biomedical HIV prevention interventions remains limited.

**Objective**: This systematic review aims to synthesize evidence on how school-based programs implement, deliver and achieve outcomes in HIV prevention interventions involving pre-exposure prophylaxis, condom and HIV testing for adolescent girls and young women in sub-Saharan Africa.

**Materials and Methods:** This review will follow the Preferred Reporting Items for Systematic Reviews and Meta-Analyses Protocols guidelines. Eligible studies will include randomized and non-randomized trials and qualitative research published between January 2015 and December

2025. Databases to be searched include PubMed, Embase, PsycINFO, CINAHL, African Index Medicus, and ProQuest. Two reviewers will independently screen titles, abstracts, and full texts, extract data, and assess risk of bias using appropriate tools. Quantitative data will be synthesized using meta-analysis where feasible, while qualitative findings will undergo thematic synthesis. Outcomes of interest include the feasibility, accessibility, and effectiveness of school-based HIV prevention interventions.

**Discussion**: This review will provide a comprehensive synthesis of evidence on biomedical HIV prevention interventions delivered in school settings for adolescent girls and young women in sub-

51	Saharan Africa. Findings will highlight implementation process, barriers, facilitators and equity 52	considerations.

53

54

55	Trial registration number: PROSPERO CRD420251249595

56

# Introduction

Global progress in human immunodeficiency virus (HIV) prevention has accelerated in recent years, with the year 2024 recording a 40% reduction in incidence compared to 2010. However, an estimated 1.3 million people acquired HIV in 2024, nearly three times the global target of fewer than 370,000 new infections by 2025 (1). Sub-Saharan Africa accounted for half of all new infections, even as the region experienced the steepest decline globally, a 56% decrease in incidence over the same period (1).

Adolescent girls and young women (AGYW) remain disproportionately affected. HIV incidence among AGYW is more than three times higher than that of their male peers (1). Although global targets aimed to reduce new infections among AGYW to less than 50,000 by 2025, an estimated 210,000 AGYW acquired HIV in 2024 (1). In eastern and southern Africa, the estimated number of new HIV infections among AGYW was 140,000, and in western and central Africa it was 31,000 (1)**.** This persistent vulnerability reflects deep-rooted gender inequalities, power imbalances, restrictive social norms, stigma, discrimination, criminalization, violence and socioeconomic inequalities across sub-Saharan Africa, which collectively limit AGYW’s ability to negotiate safer sex, access services, and exercise sexual autonomy (1,2).

Despite the availability of effective prevention tools, uptake is inadequate. Condom use, one of the most cost-effective HIV prevention strategies, remains suboptimal due to stigma, limited access, gendered power dynamics, and scarcity of youth-friendly service environments (3–5). Likewise, biomedical HIV prevention options such as pre-exposure prophylaxis (PrEP), condom distribution and HIV testing, have expanded the prevention landscape, yet their uptake among

AGYW is suboptimal, especially within traditional facility-based models (1).

Schools represent one of the most consistent and scalable contact points for reaching AGYW in sub-Saharan Africa. They offer access to large cohorts of adolescents in structured settings and can serve as effective entry points for integrated sexual and reproductive health (SRH) interventions. Evidence demonstrates that educational programmes can enhance HIV-related knowledge, build agency, and promote protective behaviours among adolescents (6,7). However, curriculum-based HIV education alone has shown limited effectiveness in reducing HIV incidence or maintaining behaviour change without complementary access to prevention services (8,9).

Emerging research suggests that schools may also be promising delivery sites for biomedical HIV prevention interventions. Studies have reported higher PrEP uptake in school-based or school-linked delivery models compared to community or clinic-based approaches (10). Qualitative research further indicates that comfort, accessibility, trust, and peer support facilitate PrEP initiation among AGYW in school environments (11). This suggests that integrating biomedical prevention within school settings may help overcome many of the barriers that limit AGYW’s access to facility-based services.

Existing reviews have focused largely on sexual and reproductive health educationa, abstinence, or broader behavioural interventions, with limited attention to school-based biomedical or service-oriented HIV prevention initiatives and their implementation outcomes (8,12–14).

This systematic review will address this gap by synthesizing quantitative and qualitative evidence to understand what works, what is feasible, and what barriers and facilitators influence the implementation of school-based HIV prevention initiatives, including PrEP, condom, and HIV testing for AGYW in sub-Saharan Africa. The findings will inform programme design, implementation, and scale-up of efforts aimed at reducing HIV incidence among AGYW.

## Objective

To synthesize evidence on how school-based programs are implemented, delivered, and achieve outcomes in HIV prevention interventions (involving PrEP, condom and HIV testing) for AGYW in sub-Saharan Africa.

### Review questions

1. What is known about the feasibility of implementing PrEP, condom or HIV testing interventions in school settings in sub-Saharan Africa?
2. What evidence exists on the accessibility of such interventions among AGYW?
3. How effective are these interventions at improving HIV prevention outcomes?

# Materials and Methods

This protocol was developed using the Preferred Reporting Items for Systematic reviews and Meta-Analyses Protocols (PRISMA-P) checklist.

## Eligibility criteria

Selected studies will be based on the eligibility criteria outlined in Table 1 below:

### Table 1 Study Eligibility criteria

| **Component** | **Include** | **Exclude** |
| --- | --- | --- |
| **Population** | AGYW, aged 10-24 years, enrolled in schools in sub-Saharan Africa | Studies focused exclusively on boys or adult women,  Out-of-school youth  Studies not disaggregating AGYW data  Studies conducted outside sub-  Saharan Africa |
| **Intervention** | School-based HIV prevention programs that include at least one of the following: PrEP education/delivery, condom promotion/distribution, or HIV testing/counselling (in-school or linked to external services) | No intervention,  Interventions delivered solely in community or clinic settings without school linkage  Interventions limited to general life skills or abstinence-only programs without biomedical or service components. |
| **Outcomes** | Feasibility, accessibility,  effectiveness  Biological/behavioural outcomes | Studies reporting only process indicators without implementation or effectiveness outcomes |
| **Study design** | Randomized-controlled trials,  Cluster-randomized trials  Quasi-experimental studies,  Cohort/longitudinal, implementation studies, mixed-methods studies, qualitative studies | Editorials, commentaries, reviews |
| **Context** | Formal educational institutions (e.g., public, private, faith-based, | Non-school settings (e.g. community centres, Clinics, households) |
| **Component** | **Include** | **Exclude** |
|  | vocational schools) in sub-Saharan Africa |  |
| **Time Frame** | Studies published between January  2015 and December 2025 | Studies published before 2015 |
| **Language** | English | Non-English publications |

## Information sources

### Search strategy

We will search multiple online databases to identify peer-reviewed articles and grey literature. The following databases will be searched:

- PubMed through the PubMed interface using MeSH terms and keywords
- Embase via Elsevier using Emtree Terms and keywords
- PsycINFO via EBSCOhost using APA Thesaurus and keywords
- CINAHL via EBSCOhost using CINAHL Subject Headings and keywords
- African Index Medicus via WHO AIM portal using keywords
- WHO Global Health Library via the WHO portal
- Grey literature: WHO, UNAIDS, UNICEF, PEPFAR

The search will be limited to studies published from January 1, 2015, up to the date of the final search. Only English-language search terms will be used to construct the queries. Table 2 presents an example of the search strategy to be used in PubMed.

#### Table 2. Search strategy in PubMed

| **Search** | **Search term** |
| --- | --- |
| 1 | “Schools” [MeSH] OR school-based [tw] OR school* [tw] OR education* [tw] OR classroom*[tw] OR academic* [tw] OR campus* [tw] OR secondary school [tw] OR high school [tw] |
| 2 | “HIV infections/prevention and control” [MeSH] OR “HIV Testing” [MeSH] OR  “Condoms” [MeSH] OR “Pre-Exposure Prophylaxis” [MeSH] OR “Sexual Health/education” [MeSH] OR “Student Health Services” [MeSH] OR HIV prevention[tw] OR HIV testing[tw] OR PrEP [tw] |
| 3 | “Adolescent” [MeSH] OR “Young Adult” [MeSH] OR “Female” [MeSH] OR  “Students” [MeSH] OR adolescent girls[tw] OR young women [tw] OR AGYW [tw] OR teen* [tw] OR youth [tw] OR student* [tw] |
| 4 | “sub-Saharan Africa”[tw] OR “Africa South of the Sahara” [MeSH] OR Africa [tw]  OR Angola [tw] OR Benin[tw] OR Botswana [tw] OR “Burkina Faso"[tw] OR  Burundi [tw] OR Cameroon [tw] OR “Cape Verde” [tw] OR Cabo Verde[tw] OR  “Central African Republic” [tw] OR Chad [tw] OR Comoros [tw] OR Congo [tw] OR  “Democratic Republic of the Congo” [tw] OR Djibouti [tw] OR “Equatorial Guinea”  [tw] OR Eritrea [tw] OR Ethiopia [tw] OR Gabon [tw] OR Gambia [tw] OR Ghana [tw] OR Guinea [tw] OR “Guinea Bissau” [tw] OR “Ivory Coast” [tw] OR “Cote d’Ivoire” [tw] OR Kenya [tw] OR Lesotho [tw] OR Liberia [tw] OR Madagascar [tw]  OR Malawi [tw] OR Mali [tw] OR Mauritania [tw] OR Mauritius [tw] OR  Mozambique [tw] OR Namibia [tw] OR Niger [tw] OR Nigeria [tw] OR Rwanda [tw]  OR “Sao Tome” [tw] OR Senegal [tw] OR Seychelles [tw] OR “Sierra Leone” [tw] OR  Somalia [tw] OR “South Africa” [tw] OR “South Sudan” [tw] OR Sudan [tw] OR  Swaziland [tw] OR Eswatini[tw] OR Tanzania [tw] OR Togo [tw] OR Uganda [tw] OR  “Western Sahara” [tw] OR Zaire [tw] OR Zambia [tw] OR Zimbabwe [tw] |
| 5 | #1 AND #2 AND #3 AND #4 |
| 6 | #5 Limits: 01/01/2015 to 31/12/2025, studies in English |

## Study records

### Data management

All records identified through database searches will be imported into Zotero for reference management. Full-text articles will be downloaded and stored locally, organized by first author surname and year of publication. Extracted data, reviewer decisions, and synthesis outputs will be archived across platforms to ensure transparency and reproducibility.

### Study selection and data extraction

Title/abstract screening and full-text review will be conducted using Rayyan, a web-based tool designed to facilitate blinded and collaborative screening. Two reviewers will independently screen each record against the predefined eligibility criteria. Disagreements will be resolved through discussion or adjudication by a third reviewer. Full-text screening will follow the same dual-review process within Rayyan, with exclusion reasons documented systematically.

Studies meeting the eligibility criteria will undergo data extraction, which will be conducted independently by two reviewers using a structured template aligned with the review’s objectives. Conflicts will be discussed and resolved between the two reviewers, and where resolution cannot be reached, a third reviewer will be involved to decide. RevMan will be used to facilitate data extraction, risk of bias assessment and data synthesis.

The reference lists of all included studies will be screened to identify additional eligible studies not captured through the database search.

When relevant data are missing or unclear, the corresponding author of the article will be contacted to request additional information.

**Fig 1: PRISMA flow diagram**

### 158 Data items

#### 159 Table 3. List and definitions of all variables for which data will be sought

| **Variable** | **Definition** |
| --- | --- |
| ***Characteristics of the study*** | |
| Authors | First author and co-authors |
| Year | Year of publication |
| Country/region | Country or region within sub-Saharan Africa |
| Design | Study design |
| Setting | Type of school or educational institution |
| Sample size | Number of participants or clusters |
| Duration | Length of follow-up or intervention period |
| Funding source | Private sector or Government |
| ***Population and Implementation Variables*** | |
| Population | Adolescent and young women in sub-Saharan Africa |
| Age range | Specific age range of participants included in the study |
| Intervention | Type of intervention: condom-related, PrEP-related, HIV testing, or linkage to external services |
| Comparator | Standard curriculum, community-only interventions, no intervention, other schoolbased strategies |
| Delivery personnel | Who implemented the intervention (teachers, health workers, etc) |
| Mode of delivery | Format of intervention delivery |
| Engagement | Level of involvement of school staff, parents, community leaders or AGYW themselves |
| Barriers and facilitators | Contextual factors influencing implementation (e.g. gender norms, power dynamics, structural factors) |
| ***Main Outcome variables (high priority)*** | |
| Feasibility | Implementation, logistics, training fidelity to protocol |
| Accessibility | Acceptability, uptake, equity, privacy |

| Effectiveness | Biomedical/behavioural outcomes like HIV testing uptake, PrEP initiation, condom use |
| --- | --- |
| ***Additional outcomes (moderate priority)*** | |
| Implementation outcomes | Cost and cost-effectiveness, sustainability/maintenance |
| Knowledge and awareness | Changes in HIV -related knowledge or awareness, risk perception |
| Behavioural intentions | Intentions for condom use, PrEP, HIV testing |
| Adverse events or harm | Reported negative outcomes |

## Risk of bias assessment

Quality appraisal for randomized or cluster-randomized trials will be conducted using the Cochrane Collaboration risk-of-bias (ROB) tool and risk-of-bias tool for cluster-randomized trials (ROB2), respectively. For quasi-experimental trials and cohort studies, the risk of bias in nonrandomized trials (ROBINS-1) will be used. The CASP qualitative checklist will be used to appraise qualitative studies, and the Mixed Methods Appraisal Tool will be used for mixedmethods studies.

## Data synthesis

A descriptive synthesis of all included studies, summarizing intervention types, outcomes and other study characteristics will be presented using structured tables and narrative summaries. Quantitative and qualitative data will be analysed separately before integration.

Quantitative findings will be synthesized using meta-analysis where appropriate, or narratively when heterogeneity precludes statistical pooling. Binary outcomes will be summarized using risk ratios, odds ratios, or risk differences; continuous outcomes using mean differences or standardized mean differences, and time-to-event outcomes using hazard ratios. A random-effects model will be used as a default analytic approach, with heterogeneity assessed using the I^2^ statistic and Chisquare test. Where at least 10 studies contribute data, subgroup analyses will be conducted by intervention type, age group, and delivery setting. Meta-regression may be used to explore potential sources of heterogeneity. Sensitivity analyses will examine the influence of excluding studies at high risk of bias or those with missing data. If meta-regression is not feasible due to variability in study design or outcome measurement, findings will be presented narratively. Narrative synthesis will involve grouping studies by intervention type and outcome domain and summarizing results thematically.

Qualitative data will be synthesized thematically using inductive and deductive coding. Themes will be organized under feasibility, accessibility, and effectiveness to align with the review’s core domains. Integration will occur at the interpretive level, with narrative juxtaposition of quantitative and qualitative findings to identify convergence, divergence, and contextual insights relevant to implementation.

Publication bias will be assessed using funnel plots and statistical tests for small-study effects (e.g., Egger’s or Begg’s test), where applicable. Quantitative synthesis will be carried out in RevMan, and qualitative synthesis in Excel.

## Confidence in cumulative evidence

Two independent reviewers will use the Grading of Recommendations Assessment, Development and Evaluation (GRADE) framework to determine the certainty of evidence. Conflicts will be resolved through discussion, and a third reviewer will be involved where agreement cannot be reached.

## Ethical considerations

This is a systematic review of published and publicly available literature. Hence, it will not involve primary data collection with human participants and does not require formal ethical approval. All data will be extracted from existing studies, and no individual participant information will be identifiable. This review will be conducted in accordance with PRISMA-P guidelines to ensure transparency, reproducibility, and integrity throughout the process.

## Status and timeline of the study

Table 4 summarizes the planned timeline for screening, data extraction, analysis and expected completion of the study.

### Table 4: Study timeline

| **Review stage** | **Status at submission** | **Planned timeline** |
| --- | --- | --- |
| Protocol development | Completed | Completed prior to submission |
| Database search | Not yet started | June 2026 |
| Title/abstract screening | Not yet started | June – July 2026 |
| Full-text screening | Not yet started | August 2026 |
| Data extraction | Not yet started | September – October 2026 |
| Risk of bias assessment | Not yet started | September – October 2026 |
| Data analysis and synthesis | Not yet started | November – December 2026 |
| Report writing and dissemination | Not yet started | December 2026 – February 2027 |

# Discussion

This systematic review will synthesize evidence on school-based HIV prevention interventions targeting AGYW in sub-Saharan Africa. Implementation of these interventions is often shaped by contextual factors, including stigma, policy constraints, and resource availability.

By focusing on studies conducted within educational institutions, this review aims to clarify what works in integrating biomedical HIV prevention into school settings and to clarify implementation bottlenecks and equity gaps. The inclusion of both quantitative and qualitative components will allow for a nuanced understanding of not only outcomes but also implementation processes, barriers and facilitators.

Potential limitations include variability in study design, outcome definitions, and measurement tools, which may limit the feasibility of meta-analysis. The review will also be limited to studies published in English, which may exclude relevant evidence from francophone regions of SubSaharan Africa.

Ultimately, this review will contribute to the evidence base for equity-driven HIV prevention programming in schools, helping to inform future research, policy, and practice at reducing HIV vulnerability among AGYW in sub-Saharan Africa.

## Amendments to the study

Any important protocol amendments (such as changes to eligibility criteria, outcomes or analysis methods) will be documented in detail, including justification for the changes and will be reported in the final manuscript. All amendments will also be updated in the PROSPERO record.

# Authors’ contributions

Conceptualization: CAL-S, KT, FZ

Methodology: CAL-S, LO, KT and FZ

Writing: CAL-S and LO

Supervision: KT and FZ

Reading and approval of final manuscript: CAL-S, LO, KT, FZ

# Supporting information

**S1 Fig. PRISMA flow diagram**

**S1 Table. PRISMA-P checklist**

# References

1. UNAIDS. AIDS, crisis and the power to transform: UNAIDS Global AIDS Update 2025 [Internet]. Geneva; 2025 [cited 2025 Dec 15]. Available from: https://www.unaids.org/en/UNAIDS-global-AIDS-update-2025
2. Mojola SA, Wamoyi J. Contextual drivers of HIV risk among young African women. 2019; Available from:

http://onlinelibrary.wiley.com/doi/10.1002/jia2.25302/full

3. Mvundura M, Nundy N, Kilbourne-Brook M, Coffey PS. Estimating the hypothetical dual health impact and cost-effectiveness of the woman’s condom in selected Sub-Saharan African countries. Int J Womens Health [Internet]. 2015 May 5 [cited 2025 Nov 18];7:271–7. Available from: https://pmc.ncbi.nlm.nih.gov/articles/PMC4356704/
4. Stover J, Teng Y. The impact of condom use on the HIV epidemic. Gates Open Res [Internet]. 2021 Jun 9 [cited 2025 Nov 18];5:91. Available from:

https://pmc.ncbi.nlm.nih.gov/articles/PMC8933340/pdf/gatesopenres-5-14833.pdf

5. Aventin Á, Gordon S, Laurenzi C, Rabie S, Tomlinson M, Lohan M, et al. Adolescent condom use in Southern Africa: narrative systematic review and conceptual model of multilevel barriers and facilitators. BMC Public Health [Internet]. 2021 Dec 1 [cited 2025 Nov 18];21(1). Available from:

https://pmc.ncbi.nlm.nih.gov/articles/PMC8234649/

6. Ratnawati D, Huda MH, Mukminin MA, Widyatuti W, Setiawan A. Meta-analysis of the effectiveness of educational programs about HIV prevention on knowledge, attitude, and behavior among adolescents. Narra J [Internet]. 2024;4(2):e870. Available from: https://narraj.org/main/article/view/870
7. Niland R, Flinn C, Nearchou F. Assessing the role of school-based sex education in sexual health behaviours: a systematic review. Cogent Psychol [Internet]. 2024 [cited 2025 Nov 24];11(1). Available from: https://www.tandfonline.com/doi/full/10.1080/23311908.2024.2309752
8. Mason-Jones AJ, Sinclair D, Mathews C, Kagee A, Hillman A, Lombard C. School-based interventions for preventing HIV, sexually transmitted infections, and pregnancy in adolescents [Internet]. Vol. 2016, Cochrane Database of Systematic Reviews. John Wiley and Sons Ltd; 2016 [cited 2025 Nov 18]. Available from: https://pmc.ncbi.nlm.nih.gov/articles/PMC5461872/
9. Mahomed S, Bukusi E, Sikazwe I, Musoke P, Abdool Karim Q. A risk- and needsbased strategy for HIV prevention for adolescent girls and young women, WHO African Region. Bull World Health Organ [Internet]. 2024 Dec 1;102(12):913–5.

Available from:

https://www.ncbi.nlm.nih.gov/pmc/articles/PMC11601182/pdf/BLT.23.291160.pdf

10. Nicol E, Basera W, Turawa E, Lombard C, Funani N, Chavhalala D. Assessing PrEP initiation and adherence among high-risk, sexually active adolescents and young adults: a population-based prospective study across diverse service delivery models in a high HIV prevalent district in South Africa. AIDS Behav [Internet]. 2025 Aug 1 [cited 2025 Nov 18];29(8):2588–609. Available from:

https://pmc.ncbi.nlm.nih.gov/articles/PMC12378780/

11. Maseko B, Hill LM, Phanga T, Bhushan N, Vansia D, Kamtsendero L, et al. Perceptions of and interest in HIV pre-exposure prophylaxis use among adolescent girls and young women in Lilongwe, Malawi. PLoS One [Internet]. 2020 Jan 1 [cited 2025 Nov 18];15(1). Available from: https://pmc.ncbi.nlm.nih.gov/articles/PMC6957134/
12. Amaugo LG, Papadopoulos C, Ochieng BMN, Ali N. The effectiveness of HIV/AIDS school-based sexual health education programmes in Nigeria: A systematic review [Internet]. Vol. 29, Health Education Research. Oxford University Press; 2014 [cited 2025 Nov 18]. p. 633–48. Available from:

https://doi.org/10.1093/her/cyu002

13. Denford S, Abraham C, Campbell R, Busse H. A comprehensive review of reviews of school-based interventions to improve sexual-health [Internet]. Vol. 11, Health Psychology Review. Routledge; 2017 [cited 2025 Nov 18]. p. 33–52. Available from: https://doi.org/10.1080/17437199.2016.1240625
14. Shangase N, Kharsany ABM, Ntombela NP, Pettifor A, McKinnon LR. A

Systematic Review of Randomized Controlled Trials of School Based

Interventions on Sexual Risk Behaviors and Sexually Transmitted Infections

Among Young Adolescents in Sub-Saharan Africa. AIDS Behav [Internet]. 2021

Nov;25(11). Available from: https://link.springer.com/10.1007/s10461-021-03242-8

Figure	[Click here to access/download;Figure;S1_Fig.docx](https://www2.cloud.editorialmanager.com/pone/download.aspx?id=44134341&guid=9667f893-f398-4787-8038-a9585f9b6c2e&scheme=1)

Results of database

search

(

n

=)

Results of reference list

screening (n=)

Title/Abstract screening

n

(

=)

Full

-

text screening

(

=)

n

Eligible s

tudies

for data extraction

=)

n

(

Duplicate records

n

(

=)

E

xcluded

records (n=)

Excluded records

n

=)

(

PRISMA-P Checklist

[Click here to access/download](https://www2.cloud.editorialmanager.com/pone/download.aspx?id=44514277&guid=c0ba7a6c-eda9-46ce-a255-5f90bb1ab7e5&scheme=1)

[Supporting Information](https://www2.cloud.editorialmanager.com/pone/download.aspx?id=44514277&guid=c0ba7a6c-eda9-46ce-a255-5f90bb1ab7e5&scheme=1)

[S1_Table_PRISMA_P Checklist_v2.docx](https://www2.cloud.editorialmanager.com/pone/download.aspx?id=44514277&guid=c0ba7a6c-eda9-46ce-a255-5f90bb1ab7e5&scheme=1)
