# Supplementary material for: Integrating biomedical HIV prevention in schools: Protocol for a systematic review of interventions for adolescent girls and young women in sub-Saharan Africa
Source: PLoS One. 2026 Jul 20;21(7):e0352973. doi: 10.1371/journal.pone.0352973 (PMC13384304; doi:10.1371/journal.pone.0352973)
Supplement: Peer review history

# Peer review: Decision Letter 0

**DOI:** 10.1371/journal.pone.0352973.r001

**Authors:** Oluwaseun Badru

29 May 2026

PONE-D-26-18433

Integrating biomedical HIV prevention in schools: Protocol for a systematic review of interventions for Adolescent Girls and Young Women in Sub-Saharan Africa

PLOS One

Dear Dr. Lodonu-Senoo,

Thank you for submitting your manuscript to PLOS ONE. After careful consideration, we feel that it has merit but does not fully meet PLOS ONE’s publication criteria as it currently stands. Therefore, we invite you to submit a revised version of the manuscript that addresses the points raised during the review process.

==============================

**ACADEMIC EDITOR:**

- This proposed systematic review is crucial in the context of biomedical intervention
- Please address the reviewer comments adequately, in particular the language restriction and age range.

-->==============================

Please submit your revised manuscript by Jul 13 2026 11:59PM. If you will need more time than this to complete your revisions, please reply to this message or contact the journal office at plosone@plos.org. When you're ready to submit your revision, log on to https://www.editorialmanager.com/pone/ and select the 'Submissions Needing Revision' folder to locate your manuscript file.

Please include the following items when submitting your revised manuscript:

- A letter that responds to each point raised by the academic editor and reviewer(s). You should upload this letter as a separate file labeled 'Response to Reviewers'.
- A marked-up copy of your manuscript that highlights changes made to the original version. You should upload this as a separate file labeled 'Revised Manuscript with Track Changes'.
- An unmarked version of your revised paper without tracked changes. You should upload this as a separate file labeled 'Manuscript'.

If you would like to make changes to your financial disclosure, please include your updated statement in your cover letter. Guidelines for resubmitting your figure files are available below the reviewer comments at the end of this letter.

If applicable, we recommend that you deposit your laboratory protocols in protocols.io to enhance the reproducibility of your results. Protocols.io assigns your protocol its own identifier (DOI) so that it can be cited independently in the future. For instructions see: https://journals.plos.org/plosone/s/submission-guidelines#loc-laboratory-protocols. Additionally, PLOS ONE offers an option for publishing peer-reviewed Lab Protocol articles, which describe protocols hosted on protocols.io. Read more information on sharing protocols at https://plos.org/protocols?utm_medium=editorial-email&utm_source=authorletters&utm_campaign=protocols.

As the corresponding author, your ORCID iD is verified in the submission system and will appear in the published article. PLOS supports the use of ORCID, and we encourage all coauthors to register for an ORCID iD and use it as well. Please encourage your coauthors to verify their ORCID iD within the submission system before final acceptance, as unverified ORCID iDs will not appear in the published article. *Only*  the individual author can complete the verification step; PLOS staff *cannot*  verify ORCID iDs on behalf of authors.

We look forward to receiving your revised manuscript.

Kind regards,

Oluwaseun Abdulganiyu Badru,

Academic Editor

PLOS One

Journal Requirements:

When submitting your revision, we need you to address these additional requirements.

1. Please ensure that your manuscript meets PLOS ONE's style requirements, including those for file naming. The PLOS ONE style templates can be found at

https://journals.plos.org/plosone/s/file?id=wjVg/PLOSOne_formatting_sample_main_body.pdf and https://journals.plos.org/plosone/s/file?id=ba62/PLOSOne_formatting_sample_title_authors_affiliations.pdf

2. Your ethics statement should only appear in the Methods section of your manuscript. If your ethics statement is written in any section besides the Methods, please move it to the Methods section and delete it from any other section. Please ensure that your ethics statement is included in your manuscript, as the ethics statement entered into the online submission form will not be published alongside your manuscript.

3. Please provide a complete Data Availability Statement in the submission form, ensuring you include all necessary access information or a reason for why you are unable to make your data freely accessible. If your research concerns only data provided within your submission, please write "All data are in the manuscript and/or supporting information files" as your Data Availability Statement.

4. Please ensure that you refer to Figure 1 in your text as, if accepted, production will need this reference to link the reader to the figure.

5. We note you have included a table to which you do not refer in the text of your manuscript. Please ensure that you refer to Table 3 in your text; if accepted, production will need this reference to link the reader to the Table.

6. We notice that your supplementary figures are uploaded with the file type 'Figure'. Please amend the file type to 'Supporting Information'. Please ensure that each Supporting Information file has a legend listed in the manuscript after the references list.

[Note: HTML markup is below. Please do not edit.]

Reviewers' comments:

Reviewer's Responses to Questions

**Comments to the Author**

1. Does the manuscript provide a valid rationale for the proposed study, with clearly identified and justified research questions?

The research question outlined is expected to address a valid academic problem or topic and contribute to the base of knowledge in the field.

Reviewer #1: Yes

Reviewer #2: Yes

**********

2. Is the protocol technically sound and planned in a manner that will lead to a meaningful outcome and allow testing the stated hypotheses?

The manuscript should describe the methods in sufficient detail to prevent undisclosed flexibility in the experimental procedure or analysis pipeline, including sufficient outcome-neutral conditions (e.g. necessary controls, absence of floor or ceiling effects) to test the proposed hypotheses and a statistical power analysis where applicable. As there may be aspects of the methodology and analysis which can only be refined once the work is undertaken, authors should outline potential assumptions and explicitly describe what aspects of the proposed analyses, if any, are exploratory.

Reviewer #1: Yes

Reviewer #2: Yes

**********

3. Is the methodology feasible and described in sufficient detail to allow the work to be replicable?

Reviewer #1: No

Reviewer #2: Yes

**********

4. Have the authors described where all data underlying the findings will be made available when the study is complete?

The PLOS Data policy requires authors to make all data underlying the findings described in their manuscript fully available without restriction, with rare exception, at the time of publication. The data should be provided as part of the manuscript or its supporting information, or deposited to a public repository. For example, in addition to summary statistics, the data points behind means, medians and variance measures should be available. If there are restrictions on publicly sharing data—e.g. participant privacy or use of data from a third party—those must be specified.

Reviewer #1: No

Reviewer #2: No

**********

5. Is the manuscript presented in an intelligible fashion and written in standard English?

*PLOS ONE*  does not copyedit accepted manuscripts, so the language in submitted articles must be clear, correct, and unambiguous. Any typographical or grammatical errors should be corrected at revision, so please note any specific errors here.

Reviewer #1: Yes

Reviewer #2: Yes

**********

6. Review Comments to the Author

Please use the space provided to explain your answers to the questions above and, if applicable, provide comments about issues authors must address before this protocol can be accepted for publication. You may also include additional comments for the author, including concerns about research or publication ethics.

You may also provide optional suggestions and comments to authors that they might find helpful in planning their study.

(Please upload your review as an attachment if it exceeds 20,000 characters)

Reviewer #1: The manuscript presents a generally relevant and timely rationale for examining the integration of biomedical HIV prevention interventions within school settings for adolescent girls and young women, particularly given the ongoing burden of HIV in this population and the importance of school platforms for prevention delivery. However, the justification for the proposed research questions and eligibility criteria would benefit from further refinement. In particular, the inclusion of a broad age range (10–24 years) is not fully aligned with a school-based intervention focus and introduces substantial heterogeneity across distinct developmental, educational, and service-access stages. Early adolescents (10–14 years), mid-to-late adolescents (15–19 years), and young adults (20–24 years) differ meaningfully in terms of school exposure, sexual and reproductive health needs, and relevance to biomedical HIV prevention interventions. As currently framed, the criteria risk conflating populations for whom school-based biomedical integration may be variably applicable or minimally relevant, particularly at the younger end of the spectrum. The authors should either provide a clearer justification for maintaining this broad age range, including a plan for stratified analysis by age/education stage, or consider narrowing the population to better reflect school-attending secondary school-aged adolescents, where the intervention context is most coherent.

Reviewer #2: The protocol addresses a highly relevant and timely public health issue. The focus on school-based biomedical HIV prevention for adolescent girls and young women in sub-Saharan Africa addresses an important evidence gap, particularly given the continued disproportionate burden of HIV in this population. It is also well-structured and aligned with PRISMA-P guidelines, with notable strengths including the inclusion of both quantitative and qualitative evidence, the use of established risk-of-bias tools, and the focus on implementation outcomes.

To further strengthen the manuscript, the following suggestions may be considered:

Introduction/ Methods

1. Some terms such as ‘biomedical HIV prevention’ and ‘implementation outcomes’ could be defined more clearly to improve clarity and help readers better understand the scope of the review.

Methods

1. The outcomes section could benefit from further clarification. While biological and behavioural outcomes appear to be excluded in the eligibility criteria, they are later mentioned under effectiveness outcomes. Clarifying how effectiveness will be assessed would improve consistency.

2. It would be useful to briefly explain the rationale for limiting the review to studies published between 2015 and 2025, particularly whether this relates to important policy developments such as WHO PrEP guideline updates or the SDG era.

3. The search strategy could be strengthened. Using “Africa” alone may retrieve studies outside sub-Saharan Africa.

4. The risk of bias section could be strengthened by specifying which appraisal tools will be used for each study design and how disagreements between reviewers will be resolved.

5. The data synthesis section would benefit from a little more detail regarding which outcomes will be pooled, the effect measures to be used, how heterogeneity will be assessed, and under what conditions meta-analysis will be conducted

6. Restricting the review to English-language publications may exclude relevant studies from francophone and lusophone countries within sub-Saharan Africa. The authors may wish to consider including studies published in other languages where feasible, using available translation tools or multilingual support to minimize language bias and improve the comprehensiveness of the review.

**********

7. PLOS authors have the option to publish the peer review history of their article (what does this mean?). If published, this will include your full peer review and any attached files.

If you choose “no”, your identity will remain anonymous but your review may still be made public.

**Do you want your identity to be public for this peer review?**  For information about this choice, including consent withdrawal, please see our Privacy Policy.

Reviewer #1: **Yes:** Pelisa Nongena

Reviewer #2: No

**********

[NOTE: If reviewer comments were submitted as an attachment file, they will be attached to this email and accessible via the submission site. Please log into your account, locate the manuscript record, and check for the action link "View Attachments". If this link does not appear, there are no attachment files.]

To ensure your figures meet our technical requirements, please review our figure guidelines: https://journals.plos.org/plosone/s/figures

You may also use PLOS’s free figure tool, NAAS, to help you prepare publication quality figures: https://journals.plos.org/plosone/s/figures#loc-tools-for-figure-preparation.

NAAS will assess whether your figures meet our technical requirements by comparing each figure against our figure specifications.

Attachment Submitted filename: PONE-D-26-18433_reviewed.docx

# Author comment: Author response to Decision Letter 1

**DOI:** 10.1371/journal.pone.0352973.r002

16 Jun 2026

Dear Academic Editor and Reviewers,

We sincerely thank the Academic Editor and Reviewers for their thoughtful and constructive comments. We appreciate the positive assessment of the relevance and potential contribution of this protocol. We have carefully revised the manuscript to address all comments and believe that these revisions have strengthened the clarity, transparency, and reproducibility of the proposed review. Below, we provide a detailed point-by-point response.

Academic Editor

Comment:

Please address the reviewer comments adequately, in particular the language restriction and age range.

Response:

We thank the Academic Editor for this recommendation. We have addressed all comments raised by the reviewers. We have provided additional justification for retaining the age range of 10–24 years and have incorporated plans for subgroup analyses by age category (10–14, 15–19, and 20–24 years) where sufficient data are available. We have expanded the discussion of language restrictions and explicitly acknowledge the potential for language bias resulting from the inclusion of English-language publications only. We have also explained that resource constraints preclude reliable screening and extraction of non-English studies.

Journal requirements

Comment 1:

Please ensure that the manuscript meets PLOS ONE style requirements, including those for file naming.

Response:

The manuscript has been reviewed and revised to ensure compliance with PLOS ONE formatting and style requirements.

Comment 2:

Your ethics statement should only appear in the Methods section of your manuscript. If your ethics statement is written in any section besides the Methods, please move it to the Methods section and delete it from any other section. Please ensure that your ethics statement is included in your manuscript, as the ethics statement entered into the online submission form will not be published alongside your manuscript.

Response:

The ethics statement has been retained only within the Methods section and removed from any other sections of the manuscript.

Comment 3:

Please provide a complete Data Availability Statement in the submission form, ensuring you include all necessary access information or a reason for why you are unable to make your data freely accessible. If your research concerns only data provided within your submission, please write "All data are in the manuscript and/or supporting information files" as your Data Availability Statement.

Response:

The Data Availability Statement has been revised to indicate that all data are in the manuscript and/or supporting information files.

Comment 4:

Please ensure that you refer to Figure 1 in your text as, if accepted, production will need this reference to link the reader to the figure.

Response:

An explicit reference to Figure 1 has been added within the Study Selection section.

Comment 5:

We note you have included a table to which you do not refer in the text of your manuscript. Please ensure that you refer to Table 3 in your text; if accepted, production will need this reference to link the reader to the Table.

Response:

An explicit reference to Table 3 has been added within the Data Items section.

Comment 6:

We notice that your supplementary figures are uploaded with the file type 'Figure'. Please amend the file type to 'Supporting Information'. Please ensure that each Supporting Information file has a legend listed in the manuscript after the references list.

Response:

We thank the editorial team for noting the file type issue. The figure in question was mistakenly uploaded as Supporting Information. We have corrected this by re uploading it as a main text figure and removing it from the Supporting Information files.

REVIEWER #1

Comment:

The manuscript presents a generally relevant and timely rationale for examining the integration of biomedical HIV prevention interventions within school settings for adolescent girls and young women, particularly given the ongoing burden of HIV in this population and the importance of school platforms for prevention delivery. However, the justification for the proposed research questions and eligibility criteria would benefit from further refinement. In particular, the inclusion of a broad age range (10–24 years) is not fully aligned with a school-based intervention focus and introduces substantial heterogeneity across distinct developmental, educational, and service-access stages. Early adolescents (10–14 years), mid-to-late adolescents (15–19 years), and young adults (20–24 years) differ meaningfully in terms of school exposure, sexual and reproductive health needs, and relevance to biomedical HIV prevention interventions. As currently framed, the criteria risk conflating populations for whom school-based biomedical integration may be variably applicable or minimally relevant, particularly at the younger end of the spectrum. The authors should either provide a clearer justification for maintaining this broad age range, including a plan for stratified analysis by age/education stage, or consider narrowing the population to better reflect school-attending secondary school-aged adolescents, where the intervention context is most coherent.

Response:

We thank the reviewer for this thoughtful observation. We have retained the 10–24-year age range because it aligns with global definitions of adolescents and young people used by WHO and UNAIDS, and because school-based and school-linked HIV prevention interventions in sub-Saharan Africa frequently reach individuals across this span, including early adolescents in upper primary school, secondary school students, and young adults enrolled in post-secondary or vocational institutions.

However, we agree that developmental and educational stages differ meaningfully within this range. To address concerns regarding heterogeneity, we have revised the Data Synthesis section to specify that subgroup analyses will be conducted by age category (10–14 years, 15–19 years, and 20–24 years) where sufficient studies are available. This approach preserves conceptual coherence while allowing us to capture the full range of school-attending populations targeted by HIV prevention programs in the region.

REVIEWER #2

Introduction/Methods

Comment 1:

Some terms such as ‘biomedical HIV prevention’ and ‘implementation outcomes’ could be defined more clearly to improve clarity and help readers better understand the scope of the review.

Response:

We appreciate this helpful suggestion. We have clarified these terms within the Methods section. Specifically, we added an operational definition stating that, for the purposes of this review, biomedical HIV prevention interventions refer to school-based interventions that provide, deliver or facilitate access to PrEP, condoms or HIV testing services. We also expanded the description of implementation-related outcomes, including feasibility, accessibility and effectiveness domains to improve clarity regarding the scope of the review.

Methods

Comment 1:

The outcomes section could benefit from further clarification. While biological and behavioural outcomes appear to be excluded in the eligibility criteria, they are later mentioned under effectiveness outcomes. Clarifying how effectiveness will be assessed would improve consistency.

Response:

Thank you for this helpful observation. We agree that the presentation of outcomes in the eligibility table may have created ambiguity. To improve clarity and ensure consistency across sections, we have revised the eligibility criteria by removing the reference to biological and behavioural outcomes from the inclusion table. These outcomes are now described exclusively under the Data Items section, where we provide operational definitions of feasibility, accessibility, and effectiveness. We also added a clarifying statement to specify that biomedical and behavioural measures (e.g., HIV testing uptake, PrEP initiation, condom use) will be assessed as effectiveness outcomes, reflecting real world effects of intervention delivery.

These revisions ensure that the eligibility criteria focus solely on the types of outcomes required for inclusion, while the Data Items and Outcomes sections clearly describe how effectiveness will be assessed.

Comment 2:

It would be useful to briefly explain the rationale for limiting the review to studies published between 2015 and 2025, particularly whether this relates to important policy developments such as WHO PrEP guideline updates or the SDG era

Response:

We have added a justification for the selected timeframe within the Search Strategy section. Specifically, we note that 2015 coincided with the adoption of the Sustainable Development Goals and WHO recommendations supporting broader use of oral PrEP among populations at substantial risk of HIV infection.

Comment 3:

The search strategy could be strengthened. Using “Africa” alone may retrieve studies outside sub-Saharan Africa.

Response:

We agree and have revised the search strategy accordingly. The standalone term “Africa” has been removed from the search strategy.

Comment 4:

The risk of bias section could be strengthened by specifying which appraisal tools will be used for each study design and how disagreements between reviewers will be resolved.

Response:

We appreciate this helpful suggestion. The Risk of Bias Assessment section has been expanded to specify the appraisal tool that will be used for each study design. We also clarify that risk-of-bias assessments will be conducted independently by two reviewers, with disagreements resolved through discussion and consultation with a third reviewer when necessary.

Comment 5:

The data synthesis section would benefit from a little more detail regarding which outcomes will be pooled, the effect measures to be used, how heterogeneity will be assessed, and under what conditions meta-analysis will be conducted

Response:

We appreciate this helpful suggestion and have revised the manuscript accordingly. We have expanded the Data Synthesis section. The revised text now specifies the types of outcomes that may be pooled, including feasibility, accessibility, and effectiveness outcomes; the effect measures that will be used for binary, continuous, and time-to-event outcomes; the methods for assessing heterogeneity using the I² statistic and Chi-square test; and the conditions under which meta-analysis, subgroup analyses, sensitivity analyses, and narrative synthesis will be conducted.

Comment 6:

Restricting the review to English-language publications may exclude relevant studies from francophone and lusophone countries within sub-Saharan Africa. The authors may wish to consider including studies published in other languages where feasible, using available translation tools or multilingual support to minimize language bias and improve the comprehensiveness of the review.

Response:

We thank the reviewer for this important observation. We acknowledge that restricting the review to English-language publications may result in exclusion of relevant evidence from francophone and lusophone countries in sub-Saharan Africa and may introduce language bias. However, resource constraints and the absence of multilingual screening and data extraction capacity preclude the reliable assessment of non-English studies. We revised the Discussion section to explicitly acknowledge the potential for language bias and its implications for the comprehensiveness of the review.

We thank the Academic Editor and Reviewers for their valuable comments. We believe the revisions have substantially strengthened the protocol and satisfactorily addressed the comments raised by the Academic Editor and Reviewers. We are grateful for the constructive feedback and consideration of our revised submission.

Attachment Submitted filename: Response to Reviewers.docx

# Editor report: Decision Letter 1

**DOI:** 10.1371/journal.pone.0352973.r003

**Authors:** Oluwaseun Badru, Oluwaseun Badru

18 Jun 2026

Integrating biomedical HIV prevention in schools: Protocol for a systematic review of interventions for Adolescent Girls and Young Women in Sub-Saharan Africa

PONE-D-26-18433R1

Dear Dr. Lodonu-Senoo,

We’re pleased to inform you that your manuscript has been judged scientifically suitable for publication and will be formally accepted for publication once it meets all outstanding technical requirements.

Within one week, you’ll receive an e-mail detailing the required amendments. When these have been addressed, you’ll receive a formal acceptance letter and your manuscript will be scheduled for publication.

An invoice will be generated when your article is formally accepted. Please note, if your institution has a publishing partnership with PLOS and your article meets the relevant criteria, all or part of your publication costs will be covered. Please make sure your user information is up-to-date by logging into Editorial Manager at Editorial Manager® and clicking the ‘Update My Information' link at the top of the page. For questions related to billing, please contact billing support.

If your institution or institutions have a press office, please notify them about your upcoming paper to help maximize its impact. If they’ll be preparing press materials, please inform our press team as soon as possible -- no later than 48 hours after receiving the formal acceptance. Your manuscript will remain under strict press embargo until 2 pm Eastern Time on the date of publication. For more information, please contact onepress@plos.org.

Kind regards,

Oluwaseun Abdulganiyu Badru,

Academic Editor

PLOS One

# Editor report: Acceptance letter

**DOI:** 10.1371/journal.pone.0352973.r004

**Authors:** Oluwaseun Badru, Oluwaseun Badru

PONE-D-26-18433R1

PLOS One

Dear Dr. Lodonu-Senoo,

I'm pleased to inform you that your manuscript has been deemed suitable for publication in PLOS One. Congratulations! Your manuscript is now being handed over to our production team.

At this stage, our production department will prepare your paper for publication. This includes ensuring the following:

* All references, tables, and figures are properly cited

* All relevant supporting information is included in the manuscript submission,

* There are no issues that prevent the paper from being properly typeset

You will receive further instructions from the production team, including instructions on how to review your proof when it is ready. Please keep in mind that we are working through a large volume of accepted articles, so please give us a few days to review your paper and let you know the next and final steps.

Lastly, if your institution or institutions have a press office, please let them know about your upcoming paper now to help maximize its impact. If they'll be preparing press materials, please inform our press team within the next 48 hours. Your manuscript will remain under strict press embargo until 2 pm Eastern Time on the date of publication. For more information, please contact onepress@plos.org.

You will receive an invoice from PLOS for your publication fee after your manuscript has reached the completed accept phase. If you receive an email requesting payment before acceptance or for any other service, this may be a phishing scheme. Learn how to identify phishing emails and protect your accounts at https://explore.plos.org/phishing.

If we can help with anything else, please email us at customercare@plos.org.

Thank you for submitting your work to PLOS ONE and supporting open access.

Kind regards,

PLOS ONE Editorial Office Staff

on behalf of

Dr. Oluwaseun Abdulganiyu Badru

Academic Editor

PLOS One
